# Biological age in critical care: current evidence, future prospects, and clinical implications

**DOI:** 10.3389/fmed.2025.1686899

**Published:** 2025-10-28

**Authors:** Li-bing Jiang, Wen Han

**Affiliations:** Department of Emergency Medicine, The Second Affiliated Hospital, Zhejiang University School of Medicine, Zhejiang Key Laboratory of Trauma, Burn, and Medical Rescue, Zhejiang Province Clinical Research Center for Emergency and Critical Care Medicine, Hangzhou, Zhejiang, China

**Keywords:** biological age, chronological age, critical care, epigenetic clocks, clinical prediction

## Abstract

Accurate assessment of critically ill patients is essential for informing treatment decisions and predicting outcomes. While chronological age—defined by the number of years lived—is commonly used in clinical practice, it does not necessarily capture a patient’s true physiological status. In contrast, biological age, which reflects genetic, environmental, and lifestyle factors, offers a more precise indicator of overall health. Emerging evidence supports its potential as a robust predictor of mortality, intensive care readmission, and disease severity in conditions such as sepsis and respiratory failure. Notably, unlike the linear progression of chronological age, biological age can fluctuate in response to acute stress and may revert to lower levels if the patient’s condition improves. This dynamic property underscores the utility of biological age in guiding invasive procedures, refining medication strategies, and optimizing nutrition and rehabilitation. The present study provides an overview of the definitions and methods used to calculate biological age, examines its current applications in critical care, and discusses its prospective roles in intensive care unit.

## Introduction

Accurate assessment of critically ill patients is very important. Many scoring tools such as Acute Physiology and Chronic Health Evaluation (APACHE) II score used in Intensive care unit (ICU) incorporate age as a variable (1). With the increasing global life expectancy, there is a growing number of elderly critical ill patients (2). Our previous research has shown that the average age of trauma patients has significantly increased over the past two decades (3). This trend presents new challenges for intensivists. Traditionally, chronological age—defined as the number of years a person has lived—which is unaffected by genetic, environmental, or lifestyle factors, and increases rigidly and linearly over time, is an important factor affecting diagnosis and treatment decisions in critically ill patients (4). However, research has found that chronological age does not always correlate with physiological health or cellular function (5). For instance, two patients with the same chronological age might have vastly different responses to disease, trauma, or recovery interventions, suggesting that a more nuanced marker is needed.

Biological age (BA) refers to the condition of an individual’s cells, tissues, and organs, reflecting how well or poorly they are aging (6). Unlike chronological age, the BA is influenced by various genetic, environmental, and lifestyle factors (7). Recent advances in science have made it possible to measure the BA using a variety of measures, including DNA methylation patterns, telomere length, and other molecular or physiological indicators of cellular health (8). Assessing the BA may allow clinicians to better predict outcomes, tailor therapies, and manage care more effectively. In the field of critical care medicine, the number of studies focusing on the BA is limited. This review briefly described the definition and estimation methods of the BA, summarized researches reporting the value of the BA among critically ill patients and proposed potential scenarios in which the BA may play a significant role in the future development of critical care practices.

### Definition of the BA

**Figure 1:**
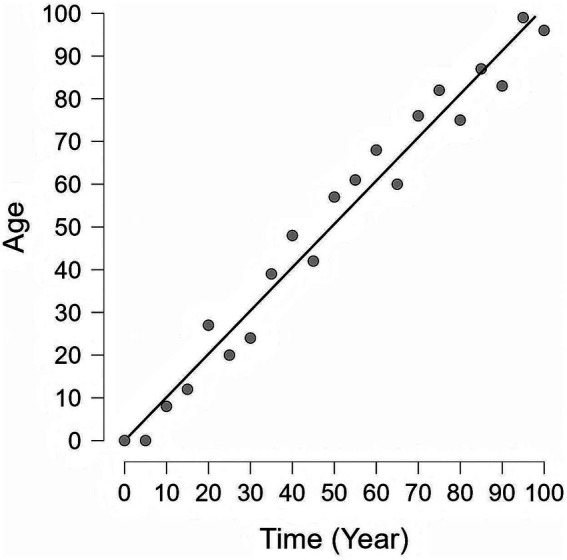
The difference between Chronological Age and Biological Age. This graph illustrates the relationship between chronological age and biological age over a lifespan (Simulated data). Black diagonal line: Chronological age, which shows a consistent and uniform rate of increase as time elapses. Gray circles: Biological age, which is affected by various internal and external factors and thus exhibits changes inconsistent with chronological age. Notably, biological age is modifiable and can potentially be reversed or stabilized through targeted interventions, emphasizing that the aging process is not purely deterministic.

The definition of the BA is currently not fully standardized. It is generally defined as the age of an individual’s cells, tissues, and organs, based on their physiological and molecular status rather than merely the number of years they have lived (9). It is shaped by a combination of intrinsic factors—most notably genetics—and extrinsic factors, which include dietary habits, physical activity levels, and exposure to environmental stressors (9). The BA can accelerate or decelerate depending on the individual’s health and lifestyle choices (Figure 1). Furthermore, within the same disease lifecycle, biological age may accelerate or decelerate in response to different treatment responses and disease trajectories (10).

### Methods of calculating the BA

There are numerous indicators and methods for calculating the BA, but the gold standard remains a topic of debate (11). Epigenetic clocks are widely used method of estimating the BA by analyzing the DNA methylation patterns. DNA methylation, a key epigenetic modification, involves the addition of methyl groups to the DNA molecule, typically at cytosine bases in CpG dinucleotides. These methylation patterns evolve over time and are influenced by a multitude of factors, including age, environment, lifestyle, and disease status. Specifically, the concept of the epigenetic clock stems from the observation that certain genomic loci undergo consistent changes in methylation as individuals age. These alterations can be harnessed to predict the BA with a remarkable degree of accuracy. The Horvath clock, developed by Steve Horvath in 2013 (12), is one of the most widely used epigenetic clocks. It estimates the BA based on DNA methylation data from over 350,000 CpG sites across the genome. Other commonly used epigenetic clocks—including the Hannum clock (13) and GrimAge (14)—focus on distinct sets of CpG sites. Additionally, telomere length, which shortens with each cellular division, has been widely used as an indicator of BA, although its reliability as a standalone measure remains a topic of debate (15).

While epigenetic clocks are accurate, their feasibility remains relatively low. Therefore, in recent years, several models for estimating the BA based on easily obtainable indicators have been proposed, including clinical biomarkers (14), metabolomics (16), microbiomics (17), functional tests (18), imaging methods (19, 20), and integrated methods (14), etc. Like PhenoAge—which is developed using clinical blood biomarkers—it offers greater accessibility and practicality for clinical use (14). The equations below describe the process of calculating PhenoAge.

While epigenetic clocks are accurate, their feasibility remains relatively low. Therefore, in recent years, several models for estimating the BA based on easily obtainable indicators have been proposed, including clinical biomarkers (14), metabolomics (16), microbiomics (17), functional tests (18), imaging methods (19, 20), and integrated methods (14), etc. Like PhenoAge—which is developed using clinical blood biomarkers—it offers greater accessibility and practicality for clinical use (14). The equations below describe the process of calculating PhenoAge.


$$10-\text{year mortality risk}=1-\mathrm{EXP}(\begin{array}{l} -\mathrm{EXP}[\text{linear predictor}]\\ {}*1.51714/0.0076927 \end{array})$$

The linear predictor = −19.907 + Albumin (g/L) × −0.0336 + creatinine (umol/L) × 0.0095 + glucose (mmol/L) × 0.1953 + Ln [C-reactive protein (mg/dL)] × 0.0954 + lymphocyte percentage (%) × −0.012 + mean red blood cell volume (fL) × 0.0268 + red blood cell distribution width (%) × 0.3306 + alkaline phosphatase (U/L) × 0.00188 + white blood cell count (10^3^ cells/mL) × 0.0554 + chronological age (years) × 0.0804.

The linear predictor = −19.907 + Albumin (g/L) × −0.0336 + creatinine (umol/L) × 0.0095 + glucose (mmol/L) × 0.1953 + Ln [C-reactive protein (mg/dL)] × 0.0954 + lymphocyte percentage (%) × −0.012 + mean red blood cell volume (fL) × 0.0268 + red blood cell distribution width (%) × 0.3306 + alkaline phosphatase (U/L) × 0.00188 + white blood cell count (10^3^ cells/mL) × 0.0554 + chronological age (years) × 0.0804.


PhenoAge=141.50+LN[−0.00553∗LN(1−Mortality risk)]/0.09165

Currently, numerous online tools and commercial platforms are available to provide blood tests and related calculations. However, the predictive variables used in these new models are prone to being influenced by confounding factors and require correction using various mathematical models, such as Multiple Linear Regression (MLR), Principal Component Analysis (PCA), and the Klemera-Doubal method (KD), etc. (8).

Recent advances in artificial intelligence (AI) and machine learning have introduced new opportunities for refining the BA predictions (21). In a recent study, the authors found a deep learning model based on easily obtainable and low-cost face photographs can estimate the BA and enhance survival prediction in patients with cancer (22). The substantial volume of data generated by ICU patients has enabled the prediction of BA using AI. Furthermore, the extensive longitudinal data available in the ICU has also rendered the prediction of dynamic changes in BA readily achievable. Furthermore, several studies have revealed that BA may vary across different organs and physiological systems, which can mutually influence one another. Using multi-omics data, Nie et al. estimated the BA of various organs (such as the liver and kidneys) and systems (including the immune and metabolic systems). Their results demonstrated heterogeneous aging rates among organs and systems, leading to the conclusion that individuals exhibit distinct aging patterns (56). Expanding on this, Ye et al. further revealed that the age of each organ selectively affects the aging rate of several interconnected organ systems. Based on these findings, they constructed multi-organ aging networks to model such interactions (7).

### Current applications of BA in critical care

Ho et al. employed the Levine PhenoAge model (based on 9 blood biomarkers reflecting DNA methylation) to investigate BA in critically ill patients. They identified a U-shaped association between BMI and both frailty (measured via the Clinical Frailty Scale) and BA residuals (BA unexplained by chronological age), with patients having BMI < 18.5 or ≥ 40 exhibiting higher frailty and more accelerated BA; crucially, only frailty (OR = 1.30 per grade increment) and BA residuals (OR = 1.20 per 10-year increment) independently predicted mortality. In addition, they reported that PhenoAgeAccel (BA older than chronological age) was more prevalent in patients with unplanned ICU readmission (52% vs. 43%), and each 10-year increase in BA residuals was associated with a 12% higher risk of unplanned readmission (OR = 1.12) after adjusting for chronological age, comorbidities, and illness severity. Finally, they also demonstrated that BA and its residuals outperformed chronological age in discriminating hospital mortality (AUROC: 0.648/0.654 vs. 0.547); PhenoAgeAccel doubled mortality risk (unadjusted HR = 1.997) with a dose-dependent relationship persisting until a 20-year residual gap, and this association remained significant after confounder adjustment (adjusted HR = 1.386) (23–25). The adjustment is critical for applying PhenoAge in acute care settings, where patient blood tests may be skewed by acute inflammation, unlike the stable health conditions in which the original model was developed. Archana et al. conducted a comparative analysis of the PhenoAge and Hannum epigenetic age algorithms in critically ill patients with and without sepsis. Their findings revealed that only the PhenoAge model’s calculation of epigenetic age acceleration was significantly associated with sepsis and mortality outcomes, whereas the Hannum algorithm did not show such correlations (26). Additionally, Xu et al. demonstrated that higher PhenoAge was linked to an increased mortality risk in heart failure patients, further emphasizing the clinical relevance of epigenetic age acceleration (27). Martina et al. identified a distinctive epigenetic signature in immune-related genes among COVID-19 patients with ARDS, highlighting the role of epigenetic modifications in immune responses during severe infections (28). Cao et al. corroborated these findings by reporting significant epigenetic age acceleration in severe COVID-19 patients compared to healthy individuals (29).

Telomere length has emerged as another critical biomarker in the context of critical illness. Liu et al. found that shorter peripheral blood leukocyte (PBL) telomere length in critically ill patients, particularly those with sepsis, was associated with poorer survival rates and more severe ARDS (30). Yosra et al. extended these observations to critically ill COVID-19 patients with ARDS, noting that both epigenetic age acceleration and telomere attrition were linked to treatment outcomes. Notably, severe COVID-19 correlated with a significant increase in DNA methylation age, while telomere attrition did not show a significant change (31). Further supporting this, Ana reported that COVID-19 ICU patients with prolonged hospital stays, the need for invasive mechanical ventilation (IMV), or the development of fibrosis exhibited shorter telomere lengths during the first year post-discharge (32). In pediatric populations, Sören et al. observed shorter leukocyte telomeres in critically ill children admitted to the PICU (33). Conversely, Benjamin noted both shortening and lengthening of telomeres in general ICU patients, although these changes did not directly correlate with patient outcomes (34). Naara’s study reinforced the importance of telomere length by documenting its shortening in sepsis patients, thereby supporting its role as a marker of critical illness severity (35). Keyvan’s research on septic shock survivors revealed a decrease in leukocyte telomere length, although no direct correlation with organ failure was identified (36).

## The future potential applications of BA in the ICU

### Patient stratification-assessment of disease severity

BA can inform decisions about preventive strategies. For example, patients with a higher BA and associated frailty may benefit from early interventions such as physical therapy, nutritional support, and targeted vaccination to prevent complications like pneumonia. Studies have shown an association between BA and complications following major cancer surgery (37). Therefore, in prehabilitation programs, the adoption of certain evidence-based strategies proven to reduce BA may be incorporated to aim for a lower incidence of postoperative complications.

Traditional ICU scoring systems (e.g., APACHE, SAPS) primarily focus on physiological and laboratory parameters, with no consideration for interindividual variations in aging processes or baseline resilience. The integration of BA could offer a more nuanced perspective: it may help identify younger patients with substantially diminished physiological reserves, as well as older patients who retain relatively intact organ function (38–40). By leveraging physiological resilience, BA might further assist in patient prioritization, thereby facilitating the development of more personalized triage strategies. Consequently, investigating whether BA outperforms chronological age in optimizing these scoring tools represents a highly promising direction for future research. Correct patient stratification and assessment is better for resources allocation. This would be particularly beneficial during health crises like pandemics, where large numbers of patients need to be triaged and treated simultaneously (41). Relying solely on chronological age can lead to undertreatment in older adults, even when they might have better biological resilience. Biological age may offer more precise criteria for ICU admission and the allocation of scarce resources, helping identify patients who have sufficient physiological reserves to benefit substantially from critical care interventions. Several studies found the BA was associated with the severity and mortality of patients with COVID-19 (29).

### BA as a predictor of clinical outcomes and prognosis

Advanced chronological age was found to be strongly associated with poor outcomes such as severe organ failure, secondary infectious complications, intensive care utilization, ventilator days, mortality, and poor discharge disposition or loss of independent living status (long-term acute care facility, skilled nursing facility, hospice etc.) (42, 43). BA, as it more accurately reflects the functional status of cells compared to chronological age, is theoretically a stronger predictor of various adverse events and prognostic outcomes than chronological age. Previous studies have found that the BA or the difference between BA and chronological age can predict the mortality rate and ICU readmission rate of critically ill patients (23–26). Therefore, the BA should be considered as one important variable for screening of risk factors and predicting the long-term and short-term prognosis of critically ill patients. In the field of critical care medicine, relatively few studies have explored and analyzed the impact of critical illnesses themselves or different interventions on the aging process and healthspan of critically ill patients. Biological age, as a surrogate marker for the latter, provides a foundation for research in this area.

### Guiding individualized treatment

Age is a critical determinant in many intensive care treatment protocols. For example, the Extracorporeal Life Support Organization (ELSO) guidelines for adult extracorporeal cardiopulmonary resuscitation (ECPR) identify being under 70 years of age as one of the indications for ECPR (44). In the future, the BA might be a more appropriate alternative to chronological age in the above mentioned algorithm to choose invasive treatments.

Many syndromes like sepsis in the ICU are highly heterogeneous, involving diverse pathophysiological processes that vary among different patients. This may partially explain the failure of many promising treatments in critically ill patients (45). More and more studies are exploring the subphenotypes of the above-mentioned syndromes based on various indicators. These subphenotypes demonstrate divergent responses to identical therapeutic interventions in randomized controlled trials (RCTs) (45), underscoring the critical need to identify distinct subgroups. Such characterization enables the delineation of patient populations who are most likely to benefit from specific treatments, thereby facilitating precision intervention strategies. In contrast to the fixed nature of chronological age, biological age (BA) dynamically adapts to disease severity and treatment efficacy, positioning it as a robust biomarker for distinguishing syndrome trajectories and optimizing patient stratification for personalized therapy.

**Figure 2:**
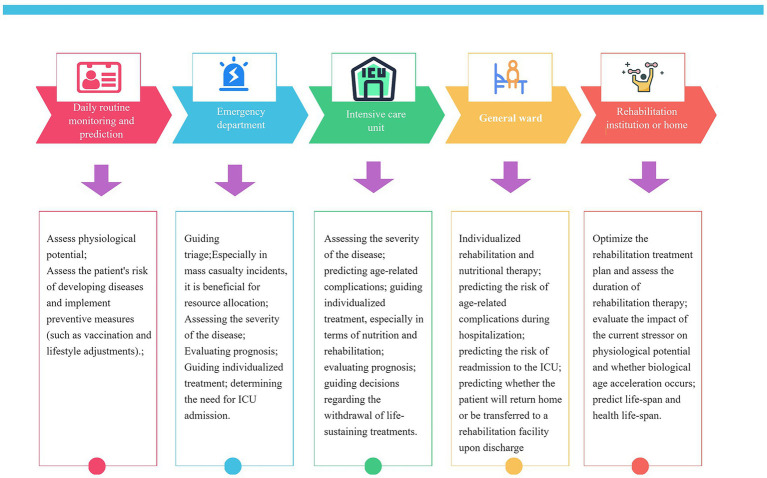
Potential value and applications of biological age at different stages in the management of critically III patients.

Jesse et al. observed that stressors including major surgery, pregnancy, and severe illnesses (e.g., COVID-19) induce an elevation in biological age (BA)—a rise that is reversed upon recovery from the stressor. This phenomenon of reversible BA has also been documented in individuals of advanced chronological age (10). Collectively, these findings underscore that BA is not a static or unidirectionally progressive metric; instead, it undergoes reversible changes across diverse timeframes, spanning from days to months. This dynamic property of BA holds substantial clinical implications. First, the efficacy of anti-aging interventions in critically ill patients could be evaluated based on the magnitude and rate of BA recovery—a principle that also extends to assessing the effectiveness of other conventional critical care therapies. For instance, Jesse et al. demonstrated that the administration of tocilizumab accelerates BA recovery in patients recovering from COVID-19 (10). Beyond treatment evaluation, BA serves as a pivotal biomarker reflecting interindividual differences in metabolism, detoxification capacity, and organ function (46), making it possible for tailoring personalized medical strategies—from drug therapies and nutritional plans to rehabilitation protocols and surgical approaches. Additionally, as advancements in intensive care lead to an increasing number of patients surviving into persistent/chronic critical illness states (47), harnessing BA’s dynamic characteristics can help identify those at higher risk of such prolonged conditions, thereby enabling timely implementation of close monitoring and more aggressive interventions to reduce the number of critically ill patients progressing to a state of chronic critical illness. Studies have demonstrated that preoperative BA is closely associated with postoperative complications in patients undergoing major oncologic surgery. Therefore, preoperative strategies aimed at reducing BA in patients with advanced biological age may help mitigate the risk of postoperative complications (36) (Figure 2).

### The implications of BA on future critical care trials

Some researchers have expressed concerns regarding the use of mortality rate as the sole endpoint in clinical trials for severe illnesses. It is equally crucial to consider other outcomes, including treatment safety, patient and family experience, accelerated recovery from critical illness, and a reduction in critical illness-associated long-term sequelae (48). The reversible nature of BA enables it to serve as a useful marker for tracking the recovery or deterioration of physiological function over time in critically ill patients. Furthermore, BA could represent a valuable endpoint in critical care medicine clinical trials—for instance, as a primary or secondary endpoint in phase II trials, or as a secondary endpoint in phase III trials involving critically ill patients or those undergoing major surgery. Additionally, clinical trials in the field of critical care medicine rarely focus on the impact of the disease itself or interventions on aging, and BA as an outcome measure can partially compensate for this gap. Additionally, traditional randomized controlled trials (RCTs) conducted in the ICU frequently rely on chronological age as either an inclusion/exclusion criterion or a stratification factor for randomization. However, two patients with the same chronological age could have vastly different health statuses. BA can better identify younger patients who are physiologically “frail” and older patients who remain physiologically robust, thus creating a more uniform study population in terms of physiological aging. Therefore, the BA might be more appropriate than chronological age. Meanwhile, BA may be more a potentially valuable factor for intervention stratification in future ICU-based trials. Critically ill patients who are biologically older than their corresponding chronological ages may be prone to benefit form more early and proactive clinical intervention measures. Thirdly, in observational studies, BA should be adjusted in screening for risk factors, evaluation of intervention efficacy, and screening for prognostic factors.

### The relationship between frailty and BA

Clinical frailty describes a state of decline in physical, physiological, and cognitive reserves, is characterized by the diminished resistance to both endogenous and exogenous stressors, which leads to an increased vulnerability of individuals to diseases (18). The prevalence of frailty increases with age and is characterized by reduced mobility, weakness, decreased muscle mass, poor nutritional status, and cognitive impairment. It is strongly associated with adverse outcomes following ICU admission, independently of and superior to chronological age (49). Frailty can be measured or assessed using various methods. Different from the traditional indicators for calculating BA, the scores used to evaluate frailty heavily weighted on assessing patient function and includes a patient’s ability to mobilize as well as inquiring about their habitual physical activity and abilities (50). Frailty can be seen as a specific expression of BA, particularly in older adults, and captures age-related decline in physiological reserve. Frailty index scores demonstrate interindividual variation among aged peers, with individual scores potentially declining while the group average increases over time, which accounts for the heterogeneity and plasticity of aging (51). Both frailty and BA are important for understanding and managing age-related health risks and promoting healthy aging. Anthony and Ho (52) conducted a single-center retrospective cohort study of 1,073 critically ill adults in Western Australia. They reported that PhenoAge and the Clinical Frailty Scale (CFS), both assessed at ICU admission, were moderately correlated and independently predictive of hospital mortality and no significant differences in their discriminative performance. The authors further observed a significant interaction between PhenoAge and frailty on mortality risk, which was most pronounced in patients without clinical frailty.

### Future Directions and Challenges of BA Application in Critical Care

The future will also witness the advancement of emerging technologies that enhance the accuracy, accessibility, and speed of BA assessments. Innovations in molecular diagnostics—such as portable or wearable devices capable of BA measurement—may facilitate the direct integration of BA testing into hospital and intensive care unit (ICU) settings (53). Such devices could generate real-time BA data for patients, enabling more timely clinical decision-making and more precise tailoring of interventions. Furthermore, these tools would provide clinicians with dynamic insights into a patient’s health trajectory, supporting the formulation of more evidence-based treatment strategies. With the help of AI-driven predictive models, BA assessments could become a routine part of precision medicine strategies, offering a personalized roadmap for managing acute illness or injury (54). Furthermore, exploring the causes of accelerated biological age is not only a future research direction but also a crucial measure to identify targets for reducing BA and delaying aging. For example, emerging evidence suggests that gut microbiota dysbiosis is associated with accelerated epigenetic aging clocks. Studies have also shown that chronic inflammation and senescence of immune cells are associated with accelerated BA. These findings provide important insights into the mechanisms of biological aging acceleration and offer promising directions for targeted interventions (55).

Despite its potential, there are several challenges to the widespread adoption of BA in critical care. First, many of the tools used to calculate BA, such as epigenetic clocks or telomere measurements, require specialized equipment and expertise, which may not be readily available in all hospitals or clinics. Additionally, BA is influenced by a variety of factors, including genetics, lifestyle, and environmental exposures, which can make it difficult to interpret in the context of acute illness. Another limitation is that the assessment of BA lacks standardized criteria. This uncertainty can impact the making of clinical decisions and hinder cross-institutional comparisons. The establishment of unified standards for BA assessment and their validation through large-scale studies are essential. Ethical considerations are also paramount. Beyond the necessity to protect patient privacy, the potential misuse of BA assessments in clinical practice, particularly in terms of resource allocation and access to care, warrants significant attention. For example, there is a risk that individuals with accelerated biological aging could be unfairly deprioritized for life-saving interventions based on assumptions about their long-term survival prospects. The other ethical issue is the misuse of BA for being declined or paying a higher premium for insurance. To address these concerns, it is crucial that clear ethical guidelines are developed to govern the use of BA in clinical practice.

## Conclusion

Biological age shows promise as a more accurate indicator of physiological status than chronological age, offering potential benefits in risk stratification, targeted therapies, and prognostic evaluations in critical care. Further research is needed to standardize methods, address ethical issues, and integrate biological age into routine practice to improve patient outcomes.

## References

[1] Tajimi K Kosugi I Okada K Kobayashi K. Effect of reduced glutathione on hemodynamic responses and plasma catecholamine levels during metabolic acidosis. Crit Care Med. (1985) 13:178–81. doi: 10.1097/00003246-198503000-00007, PMID: 3971727

[2] Rai S Brace C Ross P Darvall J Haines K Mitchell I. Characteristics and outcomes of very elderly patients admitted to intensive care: a retrospective multicenter cohort analysis. Crit Care Med. (2023) 51:1328–38. doi: 10.1097/CCM.0000000000005943, PMID: 37219961 PMC10497207

[3] Jiang L Zheng Z Zhang M. The incidence of geriatric trauma is increasing and comparison of different scoring tools for the prediction of in-hospital mortality in geriatric trauma patients. World J Emerg Surg. (2020) 15:59. doi: 10.1186/s13017-020-00340-1, PMID: 33076958 PMC7574576

[4] Akinosoglou K Schinas G Almyroudi MP Gogos C Dimopoulos G. The impact of age on intensive care. Ageing Res Rev. (2023) 84:101832. doi: 10.1016/j.arr.2022.101832, PMID: 36565961 PMC9769029

[5] Polidori MC. Aging hallmarks, biomarkers, and clocks for personalized medicine: (re) positioning the limelight. Free Radic Biol Med. (2024) 215:48–55. doi: 10.1016/j.freeradbiomed.2024.02.012, PMID: 38395089

[6] Jazwinski SM Kim S. Examination of the dimensions of biological age. Front Genet. (2019) 10:263. doi: 10.3389/fgene.2019.00263, PMID: 30972107 PMC6445152

[7] Tian YE Cropley V Maier AB Lautenschlager NT Breakspear M Zalesky A. Heterogeneous aging across multiple organ systems and prediction of chronic disease and mortality. Nat Med. (2023) 29:1221–31. doi: 10.1038/s41591-023-02296-6, PMID: 37024597

[8] Bafei S Shen C. Biomarkers selection and mathematical modeling in biological age estimation. NPJ Aging. (2023) 9:13. doi: 10.1038/s41514-023-00110-8, PMID: 37393295 PMC10314900

[9] Jackson SH Weale MR Weale RA. Biological age--what is it and can it be measured? Arch Gerontol Geriatr. (2003) 36:103–15. doi: 10.1016/s0167-4943(02)00060-2, PMID: 12849085

[10] Poganik JR Zhang B Baht GS Tyshkovskiy A Deik A Kerepesi C. Biological age is increased by stress and restored upon recovery. Cell Metab. (2023) 35:807–820.e5. doi: 10.1016/j.cmet.2023.03.015, PMID: 37086720 PMC11055493

[11] Jia L Zhang W Chen X. Common methods of biological age estimation. Clin Interv Aging. (2017) 12:759–72. doi: 10.2147/CIA.S134921, PMID: 28546743 PMC5436771

[12] Horvath S. DNA methylation age of human tissues and cell types. Genome Biol. (2013) 14:R115. doi: 10.1186/gb-2013-14-10-r115, PMID: 24138928 PMC4015143

[13] Hannum G Guinney J Zhao L Zhang L Hughes G Sadda S. Genome-wide methylation profiles reveal quantitative views of human aging rates. Mol Cell. (2013) 49:359–67. doi: 10.1016/j.molcel.2012.10.016, PMID: 23177740 PMC3780611

[14] Levine ME Lu AT Quach A Chen BH Assimes TL Bandinelli S. An epigenetic biomarker of aging for lifespan and healthspan. Aging (Albany NY). (2018) 10:573–91. doi: 10.18632/aging.101414, PMID: 29676998 PMC5940111

[15] Vaiserman A Krasnienkov D. Telomere length as a marker of biological age: state-of-the-art, open issues, and future perspectives. Front Genet. (2020) 11:630186. doi: 10.3389/fgene.2020.630186, PMID: 33552142 PMC7859450

[16] ChailurkitLOThongmungNVathesatogkitPSritaraPOngphiphadhanakulB. Biological age as estimated by baseline circulating metabolites is associated with incident diabetes and mortality. J Nutr Health Aging. (2024) 28:100032. doi: 10.1016/j.jnha.2023.100032, PMID: 38388109

[17] Wang H Chen Y Feng L Lu S Zhu J Zhao J. A gut aging clock using microbiome multi-view profiles is associated with health and frail risk. Gut Microbes. (2024) 16:2297852. doi: 10.1080/19490976.2023.2297852, PMID: 38289284 PMC10829834

[18] Ji L Jazwinski SM Kim S. Frailty and biological age. Ann Geriatr Med Res. (2021) 25:141–9. doi: 10.4235/agmr.21.0080, PMID: 34399574 PMC8497950

[19] Dorfel RP Arenas-Gomez JM Fisher PM Ganz M Knudsen GM Svensson JE. Prediction of brain age using structural magnetic resonance imaging: a comparison of accuracy and test-retest reliability of publicly available software packages. Hum Brain Mapp. (2023) 44:6139–48. doi: 10.1002/hbm.26502, PMID: 37843020 PMC10619370

[20] Li R Chen W Li M Wang R Zhao L Lin Y. LensAge index as a deep learning-based biological age for self-monitoring the risks of age-related diseases and mortality. Nat Commun. (2023) 14:7126. doi: 10.1038/s41467-023-42934-8, PMID: 37932255 PMC10628111

[21] Gialluisi A di Castelnuovo A Donati MB de Gaetano G Iacoviello L The Moli-sani Study Investigators. Machine learning approaches for the estimation of biological aging: the road ahead for population studies. Front Med (Lausanne). (2019) 6:146. doi: 10.3389/fmed.2019.00146, PMID: 31338367 PMC6626911

[22] Bontempi D Zalay O Bitterman DS Birkbak N Shyr D Haugg F. FaceAge, a deep learning system to estimate biological age from face photographs to improve prognostication: a model development and validation study. Lancet Digit Health. (2025) 7:100870. doi: 10.1016/j.landig.2025.03.002, PMID: 40345937

[23] Ho KM Morgan DJ Johnstone M Edibam C. Biological age is superior to chronological age in predicting hospital mortality of the critically ill. Intern Emerg Med. (2023) 18:2019–28. doi: 10.1007/s11739-023-03397-3, PMID: 37635161 PMC10543822

[24] Ho KM. Biological age as a predictor of unplanned intensive care readmission during the same hospitalization. Heart Lung. (2023) 62:249–55. doi: 10.1016/j.hrtlng.2023.08.010, PMID: 37611385

[25] Ho KM. Associations between body mass index, biological age and frailty in the critically ill. Obes Res Clin Pract. (2024) 18:189–94. doi: 10.1016/j.orcp.2024.05.004, PMID: 38866643

[26] Sharma-Oates A Sullivan J Pestana D dos Santos C Binnie A Lord JM. Association of Epigenetic age and Outcome in critically ill patients. Crit Care Explor. (2024) 6:e1044. doi: 10.1097/CCE.0000000000001044, PMID: 38343441 PMC10857665

[27] Xu X Xu Z. Association between phenotypic age and the risk of mortality in patients with heart failure: a retrospective cohort study. Clin Cardiol. (2024) 47:e24321. doi: 10.1002/clc.24321, PMID: 39114957 PMC11307102

[28] Bradic M Taleb S Thomas B Chidiac O Robay A Hassan N. DNA methylation predicts the outcome of COVID-19 patients with acute respiratory distress syndrome. J Transl Med. (2022) 20:526. doi: 10.1186/s12967-022-03737-5, PMID: 36371196 PMC9652914

[29] Cao X Li W Wang T Ran D Davalos V Planas-Serra L. Accelerated biological aging in COVID-19 patients. Nat Commun. (2022) 13:2135. doi: 10.1038/s41467-022-29801-8, PMID: 35440567 PMC9018863

[30] Liu S Wang C Green G Zhuo H Liu KD Kangelaris KN. Peripheral blood leukocyte telomere length is associated with survival of sepsis patients. Eur Respir J. (2020) 55:1901044. doi: 10.1183/13993003.01044-2019, PMID: 31619475 PMC7359873

[31] Bejaoui Y Humaira AF Saad M Taleb S Bradic M Megarbane A. Epigenetic age acceleration in surviving versus deceased COVID-19 patients with acute respiratory distress syndrome following hospitalization. Clin Epigenetics. (2023) 15:186. doi: 10.1186/s13148-023-01597-4, PMID: 38017502 PMC10685564

[32] Virseda-Berdices A Behar-Lagares R Martínez-González O Blancas R Bueno-Bustos S Brochado-Kith O. Longer ICU stay and invasive mechanical ventilation accelerate telomere shortening in COVID-19 patients 1 year after recovery. Crit Care. (2024) 28:267. doi: 10.1186/s13054-024-05051-6, PMID: 39113075 PMC11308640

[33] Verstraete S Vanhorebeek I van Puffelen E Derese I Ingels C Verbruggen SC. Leukocyte telomere length in paediatric critical illness: effect of early parenteral nutrition. Crit Care. (2018) 22:38. doi: 10.1186/s13054-018-1972-6, PMID: 29463275 PMC5820800

[34] Zribi B Uziel O Lahav M Mesilati SR Singer P. Telomere length changes during critical illness: a prospective, observational study. Genes (Basel). (2019) 10:761. doi: 10.3390/genes10100761, PMID: 31569793 PMC6826589

[35] Oliveira NM Rios ECS de Lima TM Victorino VJ Barbeiro H da Silva FP. Sepsis induces telomere shortening: a potential mechanism responsible for delayed pathophysiological events in sepsis survivors? Mol Med. (2017) 22:886–91. doi: 10.2119/molmed.2016.00225, PMID: 27925632 PMC5319203

[36] Razazi K Marcos E Hüe S Boyer L Adnot S Mekontso DA. Telomere shortening during human septic shock: influence of sepsis mediators, role in organ failures, and septic myocardial dysfunction. Crit Care. (2021) 25:401. doi: 10.1186/s13054-021-03818-9, PMID: 34794487 PMC8600766

[37] Dubowitz J Cooper B Ismail H Riedel B Ho KM. Associations between biological age and complications after major cancer surgery. Anaesthesia. (2025) 80:207–10. doi: 10.1111/anae.16507, PMID: 39648740

[38] Le Gall JR Lemeshow S Saulnier F. A new simplified acute physiology score (SAPS II) based on a European/north American multicenter study. JAMA. (1993) 270:2957–63. doi: 10.1001/jama.1993.03510240069035, PMID: 8254858

[39] Vazquez G Benito S Rivera R. Simplified acute physiology score III: a project for a new multidimensional tool for evaluating intensive care unit performance. Crit Care. (2003) 7:345–6. doi: 10.1186/cc2163, PMID: 12974964 PMC270708

[40] LeGall JR Loirat P Alperovitch A. APACHE II--a severity of disease classification system. Crit Care Med. (1986) 14:754–5. doi: 10.1097/00003246-198608000-00027, PMID: 3087704

[41] Dieteren CM van Hulsen MAJ Rohde KIM van Exel J. How should ICU beds be allocated during a crisis? Evidence from the COVID-19 pandemic. PLoS One. (2022) 17:e0270996. doi: 10.1371/journal.pone.0270996, PMID: 35947541 PMC9365136

[42] Vanzant EL Hilton RE Lopez CM Zhang J Ungaro RF Gentile LF. The inflammation and host response to injury investigators, advanced age is associated with worsened outcomes and a unique genomic response in severely injured patients with hemorrhagic shock. Crit Care. (2015) 19:77. doi: 10.1186/s13054-015-0788-x, PMID: 25880307 PMC4404112

[43] Hashmi A Ibrahim-Zada I Rhee P Aziz H Fain MJ Friese RS. Predictors of mortality in geriatric trauma patients: a systematic review and meta-analysis. J Trauma Acute Care Surg. (2014) 76:894–901. doi: 10.1097/TA.0b013e3182ab0763, PMID: 24553567

[44] Richardson A Tonna JE Nanjayya V Nixon P Abrams DC Raman L. Extracorporeal cardiopulmonary resuscitation in adults. Interim guideline consensus statement from the extracorporeal life support organization. ASAIO J. (2021) 67:221–8. doi: 10.1097/MAT.0000000000001344, PMID: 33627592 PMC7984716

[45] Gordon AC Alipanah-Lechner N Bos LD Dianti J Diaz JV Finfer S. From ICU syndromes to ICU subphenotypes: consensus report and recommendations for developing precision medicine in the ICU. Am J Respir Crit Care Med. (2024) 210:155–66. doi: 10.1164/rccm.202311-2086SO, PMID: 38687499 PMC11273306

[46] Palmer AK Jensen MD. Metabolic changes in aging humans: current evidence and therapeutic strategies. J Clin Invest. (2022) 132:e158451. doi: 10.1172/JCI158451, PMID: 35968789 PMC9374375

[47] Ohbe H Satoh K Totoki T Tanikawa A Shirasaki K Kuribayashi Y. J-STAD (JAPAN Sepsis treatment and diagnosis) study group, definitions, epidemiology, and outcomes of persistent/chronic critical illness: a scoping review for translation to clinical practice. Crit Care. (2024) 28:435. doi: 10.1186/s13054-024-05215-4, PMID: 39731183 PMC11681689

[48] Legrand M. Negative trials in critical care medicine and the hurdles. Lancet Respir Med. (2018) 6:e53. doi: 10.1016/S2213-2600(18)30342-4, PMID: 30303091

[49] Jung C Guidet B Flaatten H VIP study group. Frailty in intensive care medicine must be measured, interpreted and taken into account! Intensive Care Med. (2023) 49:87–90. doi: 10.1007/s00134-022-06887-8, PMID: 36205730 PMC9540068

[50] Subramaniam A Ueno R Tiruvoipati R Srikanth V Bailey M Pilcher D. Comparison of the predictive ability of clinical frailty scale and hospital frailty risk score to determine long-term survival in critically ill patients: a multicentre retrospective cohort study. Crit Care. (2022) 26:121. doi: 10.1186/s13054-022-03987-1 35505435 PMC9063154

[51] Geense W Zegers M Dieperink P Vermeulen H van der Hoeven J van den Boogaard M. Changes in frailty among ICU survivors and associated factors: results of a one-year prospective cohort study using the Dutch clinical frailty scale. J Crit Care. (2020) 55:184–93. doi: 10.1016/j.jcrc.2019.10.016, PMID: 31739088

[52] Anthony NP Ho KM. Biological age and clinical frailty scale measured at intensive care unit admission as predictors of hospital mortality among the critically ill in Western Australia: a retrospective cohort study. Acute Crit Care. (2025) 40:264–72. doi: 10.4266/acc.000200, PMID: 40494597 PMC12151723

[53] Niederberger C Vermeersch A Davidhi F Ewald CY Havenith G Goldhahn J. Wearable sweat analysis to determine biological age. Trends Biotechnol. (2023) 41:1113–6. doi: 10.1016/j.tibtech.2023.02.001, PMID: 36822913

[54] Ashiqur RS Giacobbi P Pyles L Mullett C Doretto G Adjeroh DA. Deep learning for biological age estimation. Brief Bioinform. (2021) 22:1767–81. doi: 10.1093/bib/bbaa021, PMID: 32363395 PMC8179516

[55] Torma F Kerepesi C Jókai M Babszki G Koltai E Ligeti B. Alterations of the gut microbiome are associated with epigenetic age acceleration and physical fitness. Aging Cell. (2024) 23:e14101. doi: 10.1111/acel.14101, PMID: 38414315 PMC11019127

[56] Nie C Li Y Li R Yan Y Zhang D Li T. Distinct biological ages of organs and systems identified from a multi-omics study. Cell Rep. (2022) 38:110459. doi: 10.1016/j.celrep.2022.110459, PMID: 35263580

